# Turning Down the Inhibition Effect of Silica Gels in Protein Crystallization

**DOI:** 10.1021/acsami.5c07593

**Published:** 2025-06-20

**Authors:** Lorena Pasero, Roberto Pisano, José A. Gavira, Fiora Artusio

**Affiliations:** † Department of Applied Science and Technology, 19032 Politecnico di Torino, 24 Corso Duca Degli Abruzzi, Torino 10129, Italy; ‡ Laboratorio de Estudios Cristalográficos, 16379 Instituto Andaluz de Ciencias de la Tierra (Consejo Superior de Investigaciones Científicas), Avenida de las Palmeras 4, Armilla 18100, Granada, Spain

**Keywords:** silica gel, lysozyme, protein crystallization, nucleation, methyl groups

## Abstract

Silica gels act as nucleation inhibitors and have been used to grow large protein crystals in convection-free environments. However, a large amount of protein is required to overcome the inhibition effect, and chances of successful crystallization are limited, hampering its potential benefits. In the present study, we propose the substitution of silanol groups with methylated additives to increase the hydrophobicity of the gel network, decrease the interaction between proteins and gel fibers, and tune the inhibition effect of silica gels. We observed an increased hen egg white lysozyme (HEWL) nucleation density in gels bearing a higher number of methyl groups. We used the counter-diffusion crystallization technique for our proof of concept since it does not require a fine adjustment of the supersaturation. We then moved to batch crystallization for maintaining constant supersaturation conditions in order to have comparative results. We were able to grow HEWL crystals with tailored sizes depending on the amount of hydrophobic moieties’ substitution. The modification of the gel reduced the amount of protein required to induce nucleation. This effect was attributed to the decreased adsorption of protein macromolecules on gel fibers carrying hydrophobic groups. This simple chemical modification approach may expand the use of silica gels, traditionally seen as protein nucleation inhibitors, to produce new crystalline composite materials.

## Graphical abstract

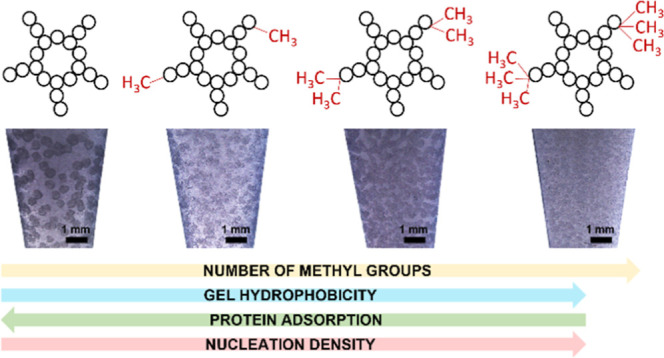

## 1 Introduction

Protein crystallization is widely employed both to determine the three-dimensional structure of proteins and to purify or administer drugs. Crystalline biologics usually possess high bioavailability, controlled release, and increased stability.^1^ However, protein crystallization still relies on trial-and-error approaches. In addition, given the complex nature of macromolecules, protein crystallization often leads to unpredictable and unreproducible results.^2^ Crystallization conditions are unique for each protein since macromolecules are extremely sensitive to aggregation and can more easily undergo precipitation or denaturation, rather than assembling into ordered arrays.

Several approaches have been proposed to promote better control over protein crystallization, such as the use of precipitants and particles,^3–6^ functionalized surfaces,^7–9^ or gels.^10^ In particular, the incorporation of gel fibers in the crystallization environment has been employed to control the crystal size,^10^ produce isotropically dyed single crystals,^11^ improve the mechanical properties^12^ and resistance to radiation damage of protein crystals,^13^ or produce composite protein crystals with modulated release profiles and increased thermal stability.^14^ Crystallization in gelled media establishes diffusion-controlled mass transport of protein macromolecules and limits sedimentation and aggregation while promoting the formation of uniform crystals and protecting them from breakage^4^ and osmotic shock.^15^ Agarose,^16,17^ silica,^18^ fluorenylmethoxycarbonyl-peptide,^14^ acrylamide,^19^ poly­(ethylene glycol) (PEG),^20^ and polysaccharides^21^ are examples of materials employed to prepare gels suitable for crystallization.

The pioneering studies by Vidal et al.^22,23^ reported two distinct groups of gels based on their effects on nucleation, i.e., nucleation promoters or inhibitors. The former property is typical of agarose gels, and it has been exploited to facilitate crystallization with reduced amounts of proteins and enhance the nucleation density, independently of the protein.^17^ The latter property characterizes silica gels, whose nucleation inhibition ability has been used to grow large single crystals suitable for crystallographic structure determination and preparation of ultrastable protein–silica composites.^18^ The decrease in nucleation rate observed in silica gels has been first attributed to the close porosity of the network, leading to compartmentalized volumes of solution containing an insufficient number of molecules to form a critical nucleus.^24^ Moreover, secondary nucleation is strongly inhibited as the collision between two growing crystals is unlikely, and convection is absent.^25^

Silica gels are produced from the polycondensation of silicic acid, which results from either the hydrolysis of siloxanes or the neutralization of sodium silicate. The polymerization reaction leads to spherical silica particles that can aggregate and originate a gel network. The particles expose unreacted silanol groups,^26^ which strongly interact with protein macromolecules via hydrogen-bonding and electrostatic interactions. The adsorption of proteins on the fibers reduces the free protein concentration in solution, thus decreasing the probability of forming nuclei, as demonstrated through small-angle neutron scattering experiments.^23^

To increase the probability of obtaining crystals in silica gels, high protein concentrations are required. However, this increases the risk of flocculation and the operative costs.^23^ To facilitate protein crystallization in silica gels, counter-diffusion crystallization (CDC) methods are commonly employed.^27^ In addition, silica gels can be chemically modified to limit protein adsorption and increase the free protein concentration in solution. For example, Vidal et al. suggested that the addition of ethoxylates promotes the crystallization of lysozyme in silica gel.^23^ Nevertheless, CDC does not lead to uniform crystal sizes, as it explores a wide range of supersaturation conditions. A deep understanding of the effect of chemically modified gels on protein nucleation is, therefore, difficult to achieve via CDC.

This work aims at investigating protein crystallization in chemically modified silica gels using additives exposing an increasing concentration of hydrophobic moieties. To the best of the authors’ knowledge, this is the first application of silica gels modified with such additives to protein crystallization. Hen egg white lysozyme (HEWL) was selected as the model protein since it is robust and easy to crystallize. Gels were prepared with different protein, precipitant, and tetramethoxysilane (TMOS) concentrations to elucidate their impact on HEWL crystallization at defined supersaturation conditions, i.e., batch conditions. Additives can effectively tune the hydrophobicity of the gel to reduce the inhibitive effect of silica gels on nucleation, by reducing the amount of protein adsorbed on the gel fibers. Furthermore, we showed the possibility of finely controlling the nucleation density according to the concentration of the additive in the gel and the number of methyl groups in the additive. The action of the modified gels on protein crystallization was related to their hydrophobicity and structure, as confirmed by contact angle (CA) and rheometry characterization.

## 2 Materials and Methods

### 2.1 Materials

Lysozyme from chicken egg white (HEWL) was purchased from Sigma-Aldrich (St. Louis, MO, USA). TMOS (99%) was purchased from Thermo Fisher Scientific (Waltham, MA, USA), while methyldiethoxysilane ( ≥96%, 1-MDEOS), dimethyldiethoxysilane (97%, 2-MDEOS), and trimethylethoxysilane (98%, 3-MEOS) were purchased from Sigma-Aldrich (St. Louis, MO, USA). The structures of TMOS, 1-MDEOS, 2-MDEOS, and 3-MEOS are depicted in Figure 1. The protein was dissolved in 50 mM sodium acetate at pH 4.5 (Sigma-Aldrich, St. Louis, MO, USA), and the resulting solution was filtered with 0.22 μm syringe filters to remove impurities. The concentration of the dissolved protein was spectrophotometrically determined at 280 nm by a Multiskan sky microplate spectrophotometer (Thermo Fisher Scientific, Waltham, MA, USA). An extinction coefficient equal to 2.65 mL cm^–1^ mg^–1^ was employed for HEWL.

**Figure 1:**
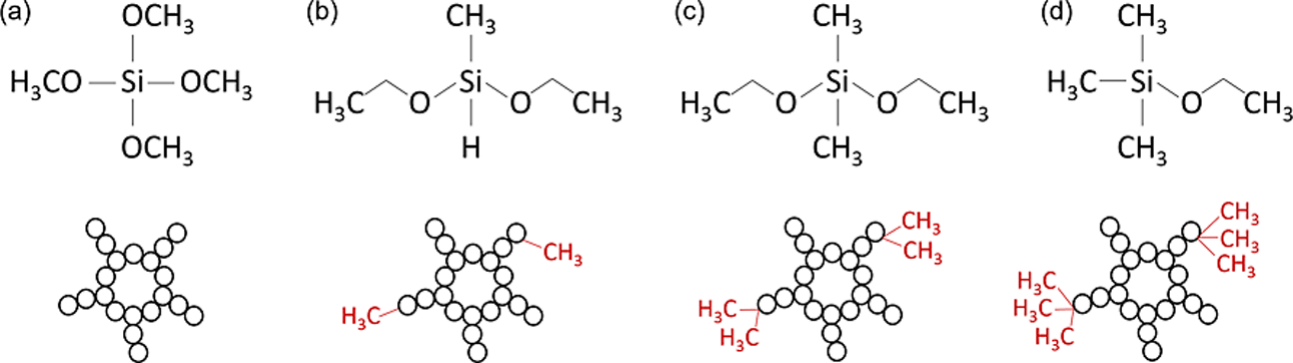
Structure of (a) TMOS, (b) 1-MDEOS, (c) 2-MDEOS, and (d) 3-MEOS and visualization of the gel structure upon substitution of the methyl groups.

### 2.2 Protein Crystallization in Silica Gel

In this study, a model protein was crystallized in silica gel by either CDC or batch crystallization methods. Table 1 lists the crystallization conditions for the selected protein, i.e., HEWL. The protein was buffered in 50 mM sodium acetate (pH 4.5) and crystallized in the presence of NaCl. The crystallization process was studied at various concentrations of TMOS and additives, i.e., 1-MDEOS, 2-MDEOS, and 3-MEOS. All the experiments were performed in duplicate, and the samples were stored at room temperature.

**Table 1:** Operative Conditions for Crystallization in the Silica Gel of HEWL

Crystallization	Protein, mg/mL	NaCl, mg/mL	TMOS, % (v/v)	AT ratio, % (v/v)
CDC	100	200	5, 10	
CDC	100	200	5	20
batch	70	50	5, 10	
batch	80	40	5, 10	
batch	50	40	10	0, 20
batch	50	50	10	0, 20
batch	70	40	10	0, 10, 20
batch	75	40	10	20
batch	80	30	10	20

#### 2.2.1 Counter-Diffusion Crystallization

CDC generally consists of three steps, i.e., the formation of the silica gel, the addition of a gel septum, and diffusion of the precipitant into the gel. To prepare the silica solution, TMOS was added to the crystallization buffer and vigorously stirred for 20 min. If present, the selected additive was added to this solution to achieve the target volume ratio between the additive and the sum of the additive and TMOS. For the sake of simplicity, from now on, we will refer to this ratio as “additive-to-TMOS ratio” (AT ratio). The appropriate volume of the silica precursor solution was then mixed with the protein in a PCR tube to obtain a final volume equal to 150 μL. Once complete gelation had been achieved, methanol produced on top of the gels was removed by rinsing them with the buffer solution. Then, each gel was covered with 75 μL of a 10% (v/v) TMOS solution to create a septum layer. After a day, the PCR vial was inserted into a 1.5 mL Eppendorf tube filled with the precipitant solution (see Figure S1 of Supporting Information). As listed in Table 1, HEWL samples were prepared with 100 mg/mL protein and 200 mg/mL NaCl. Gels with 5 or 10% (v/v) TMOS were compared in the absence of additives, whereas the effect of the additives at the AT ratio equal to 20% (v/v) was investigated, maintaining 5% (v/v) TMOS.

#### 2.2.2 Batch Crystallization

The TMOS solution was produced following the previously reported protocol, and it was mixed with predetermined amounts of protein and precipitant inside a PCR vial to obtain the desired final concentration for a total volume of 150 μL. As summarized in Table 1, HEWL was crystallized at 5 or 10% (v/v) of TMOS without additives and at different additive contents maintaining 10% (v/v) TMOS. The concentrations of HEWL and NaCl were varied between 70 and 80 mg/mL and 30–50 mg/mL, respectively. Particularly, the amounts of protein and precipitant were combined to maintain a consistent supersaturation level in the gel.

### 2.3 Microscopy

After the crystallization process had been finished, samples were analyzed by a stereo microscope (M125 C, Leica Microsystems Ltd., Wetzlar, Germany). A distinctive size was assigned to each protein crystal, i.e., the width of the (110) face. Crystals’ size was determined by measuring the size of 40 crystals through the open-source ImageJ software (NIH, Bethesda, MD, USA). The sizes are reported in the manuscript as the mean value ± standard deviation (SD).

Scanning electron microscopy (SEM) was also employed to investigate the structures of the gel and crystals. To this end, crystals were frozen in liquid nitrogen and loaded into a freeze-dryer with precooled shelves at −50 °C (LyoBeta 25, Telstar, Terrassa, Spain). Drying was then performed at 0 °C at 10 Pa for 24 h. The dried samples were extracted from the PCR vial and stuck over the surface of an aluminum stub by using a double-sided carbon tape (NEM TAPE, Nisshin LTD, Tokyo, Japan). Image acquisition was conducted by employing a Desktop SEM Phenom XL (Waltham, MA, USA) at 15 kV voltage.

### 2.4 Interaction between the Protein and Additive

The crystallization of HEWL in microbatch (MB) was performed in the absence of TMOS to investigate whether the additive and the protein interacted. To this end, a 4 μL drop was generated by mixing 2 μL of protein with 2 μL of a solution containing the precipitant either alone or combined with the additive. Particularly, a protein-to-additive mass ratio of around 12, 11.6, and 13 was selected for 1-MDEOS, 2-MDEOS, and 3-MEOS, respectively. A ratio equal to 0.4 was also examined for 1-MDEOS to study higher contents of the additive. Then, the drop was inserted into a well filled with 200 μL of paraffinic oil. For all the experiments, the HEWL concentration was varied in the range 22.5–45 mg/mL, while the NaCl concentration varied in the range 10–40 mg/mL.

### 2.5 Release Tests

As a measurement of the protein–gel interaction, the release of HEWL from the gel was measured over time. To this end, 150 μL of a solution composed of 10% (v/v) TMOS and 10 mg/mL HEWL was inserted into the wells of a 96-well plate (Costar, Kennebunk, ME, USA). After complete gelation, 100 μL of the buffer was poured over the gel. The concentration of HEWL in the buffer was spectrophotometrically measured 24, 43, 67, and 139 h after the diffusion had begun. After each sampling, the exhausted buffer was replaced with a fresh one. The procedure was repeated with gels modified with 1-MDEOS, 2-MDEOS, and 3-MEOS at AT values equal to 10 and 20% (v/v). Experiments were performed in duplicates, and the results are presented as mean ± SD.

### 2.6 Statistical Analysis

Student’s *t* test was performed through the Sigma Plot software. *p* < 0.001 and *p* < 0.05 were considered significant.

### 2.7 Characterization of the Gels

TMOS gels at 10% (v/v) concentration were prepared in 50 mM sodium acetate buffer, pH 4.5. After complete gelification had occurred, the gels were dried under a fume hood for 7 days. The procedure was repeated with gels modified with 1-MDEOS, 2-MDEOS, and 3-MEOS at AT equal to 10 and 20% (v/v). The water CA was measured via the sessile drop method using a DSA25 drop shape analyzer (Krüss Scientific, Germany) equipped with a CF03 high-speed camera with a CMOS sensor. The measurement was repeated six times per sample.

The rheological properties of 5% (v/v) TMOS gels and gels modified with 1-MDEOS, 2-MDEOS, and 3-MEOS at AT equal to 10 and 20% (v/v) prepared in 50 mM sodium acetate buffer, pH 4.5, were measured using a HAAKE Viscotester IQ Air system (Thermo Electron, Germany). A parallel-plate geometry was used, and the temperature was set at 20 °C via a Peltier temperature control system. The diameter of the upper plate was 6 cm, and the gap between the plates was 0.75 mm. The storage modulus, *G*′, was measured through oscillation frequency sweep tests. Frequency was varied between 0.1 and 10 Hz in the controlled deformation mode.

## 3 Results and Discussion

### 3.1 CDC in Silica Gel

CDC of HEWL was performed to test the action of the additives using a well-established crystallization technique for silica gels. Crystals were produced setting the HEWL concentration at 100 mg/mL, while the concentration of the precipitant diffusing into the gel was 200 mg/mL NaCl. The concentration of TMOS was 5% (v/v), since the inhibitive effect of silica at 10% (v/v) TMOS prevented nucleation, and the AT ratio was kept constant. Figure 2 displays HEWL crystals obtained by CDC in the presence of a 20% (v/v) AT ratio. As evidenced in Figure 1, the chemical structures of 1-MDEOS, 2-MDEOS, and 3-MEOS bear one, two, and three methyl groups, respectively. Upon addition to TMOS, these molecules can modify the chemistry of the silica gel by replacing silanol groups with methyl groups. Such a substitution is more pronounced as the number of available methyl groups in the additive molecule is larger. This substitution could reduce the interaction (H bonds) between HEWL and the silanol groups, thus limiting the adsorption of protein on the gel fibers.^23^ In addition, previous studies had demonstrated a favorable impact of an increase in hydrophobicity, of either surfaces^28^ or porous glasses,^29^ on the preservation of the native protein conformation. Here, we observed that the inclusion of the additives promoted nucleation, and this phenomenon was more intense as the number of methyl groups exposed to the additive increased. Such a phenomenon resulted in a progressively larger number of smaller crystals as the number of methyl groups increased. The action of the additives was attributed to the substitution of silanol groups with methyl groups in the gel. Instead, a less obvious effect was observed by comparing 2-MDEOS and 3-MEOS despite the higher number of methyl groups. This limited difference between 2-MDEOS and 3-MEOS revealed that the nucleation-inducing capacity of the gel was saturated with the 2-MDEOS additive. To better understand and characterize this behavior, we moved to batch crystallization to relate the gel-inducing behavior to fixed supersaturation conditions and performed physical characterization of the gel.

**Figure 2:**
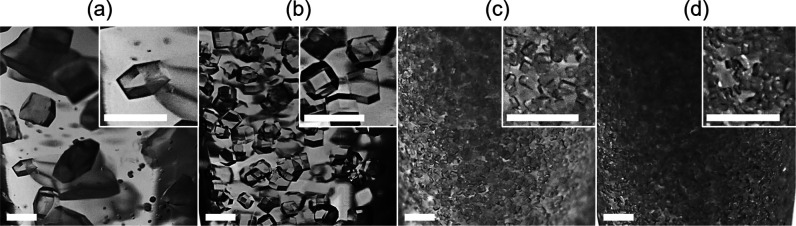
HEWL crystals produced by CDC in silica gel at 5% (v/v) TMOS and an AT ratio equal to 20% (v/v). Crystals were produced at 100 mg/mL HEWL, using a 200 mg/mL NaCl solution as a precipitant. The gels were (a) devoid of additives, (b) with 1-MDEOS, (c) with 2-MDEOS, and (d) with 3-MEOS. Scale bars refer to 500 μm.

### 3.2 Batch Crystallization in Silica Gel

Contrary to the more common CDC method, batch crystallization requires a simpler setup and guarantees better control of the crystallization outcome, ensuring a precise and known level of supersaturation throughout the experiment. However, as it is directly mixed with the precipitant, the concentration of the protein and precipitant inside the gel should be finely tuned to create supersaturation without inducing amorphous precipitation or gel flocculation. Moreover, high protein concentrations are usually required to overcome the inhibitive effect of silica gel on nucleation. To the best of our knowledge, this is the first report of HEWL crystallization in (modified) silica gels using a batch approach.

#### 3.2.1 Selection of TMOS Concentration

As reported in Figure 3, the crystallization of HEWL in silica gels prepared using different TMOS concentrations was investigated. Protein (70–80 mg/mL) and NaCl (40–50 mg/mL) concentrations were combined to maintain a comparable supersaturation level. For all the conditions, crystals appeared after 1 day, and the highest number of crystals was obtained using 70 and 50 mg/mL NaCl. At 5% (v/v) TMOS, the viscosity of the gel was lower, whereas at 10% (v/v) TMOS, the obtained gel was stiffer. This phenomenon was attributed to the inversely proportional relationship between the gelation time and the concentration of TMOS, i.e., approximately 1 day at 5% (v/v) and few hours at 10% (v/v), and to the enhanced gel connectivity at higher TMOS concentrations.^18,19,26^ At 5% (v/v), the gel was weaker than that at 10% (v/v), behaved as a viscous liquid, and could be easily collected by a pipette. Such a feature, together with the precise control of nucleation density and crystal size, represents the potential application of the use of crystal slurries prepared in 5% (v/v) TMOS gels for structural determination via X-ray free-electron laser (XFEL).

**Figure 3:**
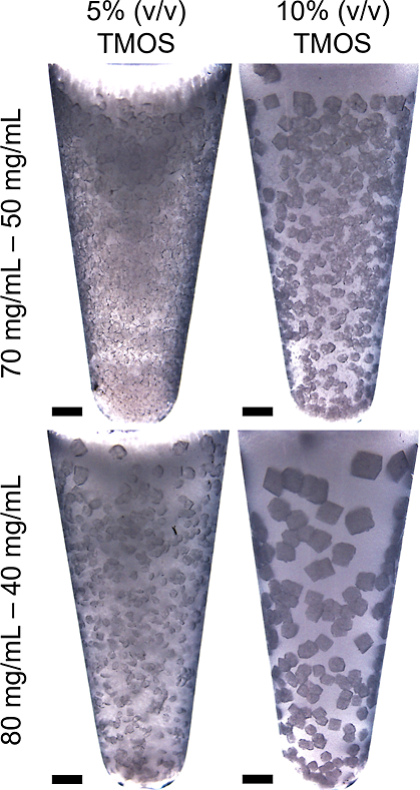
HEWL crystals produced by batch crystallization in silica gel at 5 and 10% (v/v) TMOS. Scale bars refer to 1 mm.

Interestingly, the shape of HEWL crystals seemed not affected by the TMOS content, as evidenced by the SEM images (Figure 4a). The SEM analysis shows how HEWL tetragonal crystals adapted to the silica gel during their growth, favoring the incorporation of silica fibers while keeping the habit of tetragonal crystals.

**Figure 4:**
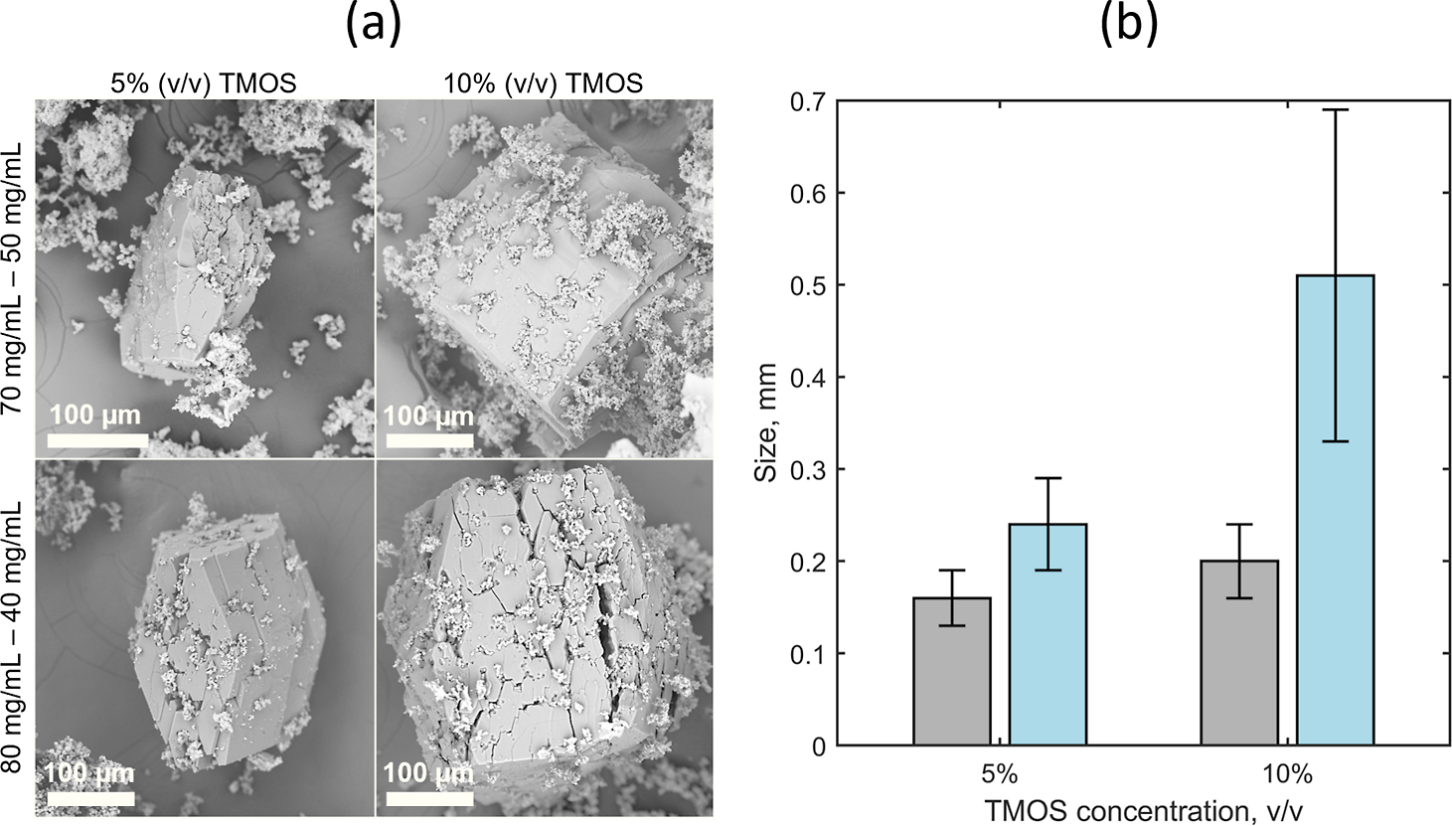
(a) SEM images and (b) size of HEWL crystals obtained at (gray) 70 mg/mL HEWL and 50 mg/mL NaCl and (blue) 80 mg/mL HEWL and 40 mg/mL NaCl. 5 and 10% (v/v) TMOS are reported.

In batch experiments, the gelation time should be shorter than the nucleation time to establish an environment with a constant supersaturation over time. This requirement is necessary to avoid supersaturation shifts during nucleation linked to the formation of the gel. Moreover, complete gelation is needed to limit convection, thus enabling correspondence between the crystal growth and the nucleation sites. Therefore, 10% (v/v) TMOS concentration was selected as the optimal one for batch crystallization as it allows gel formation in few hours, strongly reduces the nucleation density of lysozyme crystals, and is translucid, thus allowing optical microscopic observations. Fewer crystals formed at 10% (v/v) TMOS compared to 5% (v/v), as highlighted in Figure 3. A concomitant significant increase in size from 0.2 ± 0.04 mm to 0.51 ± 0.18 mm was also observed for crystals produced at 80 and 40 mg/mL NaCl moving from 5% to 10% (v/v) TMOS (Figure 4b).

#### 3.2.2 Effect of the Additives

To control the nucleation density and overcome the inhibition effects of 10% (v/v) TMOS gels, the three methylated additives were incorporated into the gels. The AT ratio was varied between 10 and 20% (v/v). Figure 5a presents the crystals obtained at 70 mg/mL of HEWL and 40 mg/mL of NaCl at different additive concentrations. These conditions were selected to prevent protein precipitation, which occurred at 80 mg/mL HEWL and 40 mg/mL NaCl in the presence of the additives (data not shown). For the AT ratio equal to 10% (v/v), the incorporation of the additive into the gel promoted nucleation, and this effect increased going from 1-MDEOS to 3-MEOS. The inclusion of increasingly methylated additives induced the formation of progressively smaller HEWL crystals and in a greater number. Coherently with CDC, the modification of the gel by substituting silanol groups with methyl groups reduced the amount of protein adsorbed on the gel. Thus, the inhibition effect of TMOS on nucleation was avoided thanks to the increased number of protein macromolecules free in solution, i.e., approaching the theoretical supersaturation level. A dramatic increase in the nucleation density was also observed when additives were added to TMOS at AT ratio equal to 20% (v/v). Nevertheless, the addition of such a high amount of additive resulted in the saturation of the nucleation-inducing ability of the silica gel. As reported in Figure 5a, the nucleation density was no longer related to the type of additive but rather to the presence of a generic methylated additive, independently from the number of its methyl groups.

**Figure 5:**
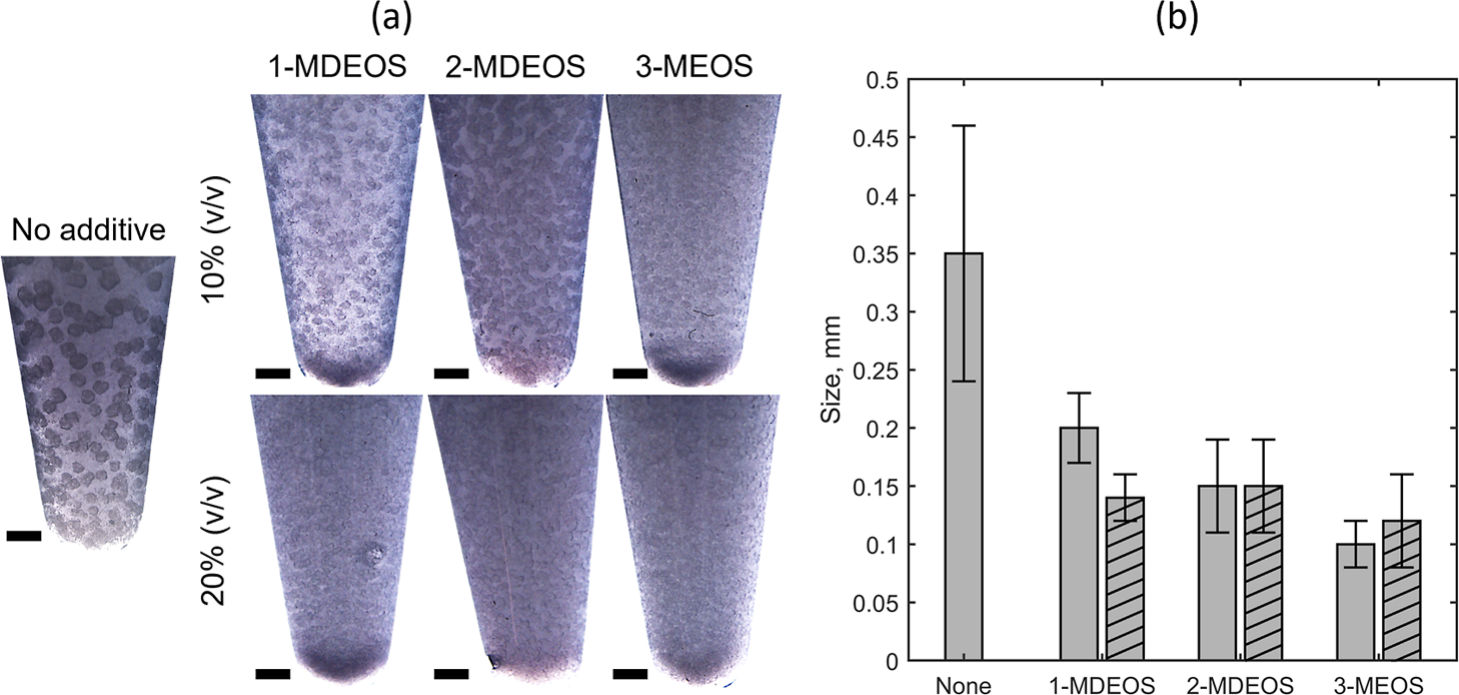
(a) HEWL crystals produced by batch crystallization in silica gel at 70 mg/mL HEWL, 40 mg/mL NaCl, and 10% (v/v) TMOS. Crystals were produced at an AT ratio equal to 10 and 20% (v/v). Scale bars refer to 1 mm. (b) Size of HEWL crystals. The filling pattern of 1-MDEOS, 2-MDEOS, and 3-MEOS bars refers to (none) 10% (v/v) AT ratio and (single diagonal) 20% (v/v) AT ratio.

The sizes of HEWL crystals produced by batch crystallization in silica gel at 70 mg/mL HEWL and 40 mg/mL NaCl are shown in Figure 5b. The biggest crystals (0.35 ± 0.11 mm) were obtained without additives, while a significant size reduction was observed in the presence of methyl groups. Considering 10% (v/v) AT ratio, the crystal size appeared to decrease as the number of methyl groups of the additive increased. Such a feature, along with the higher nucleation density, represents a clear indication of the action of methyl groups within the gel network. When the AT ratio was increased from 10% to 20% (v/v), a shift from 0.2 ± 0.03 mm to 0.14 ± 0.02 mm was observed only for 1-MDEOS, while the sizes of crystals grown in the presence of 2-MDEOS and 3-MEOS were comparable and smaller than 0.15 mm. Similar to CDC, this result disclosed that the substitution was effective until the saturation of the gel’s ability to induce protein nucleation was achieved.

As for the not-modified gel, HEWL crystals incorporated the fibers of the modified gel, preserving their tetragonal form (Figure 6). However, clusters of crystals appeared as the nucleation density increased since interpenetration among adjacent crystals occurred during the crystal growth. Indeed, at 10% (v/v) AT ratio, several single crystals could be detected, while at 20% (v/v) AT ratio, single crystals were not obtained anymore due to the extreme supersaturation conditions.

**Figure 6:**
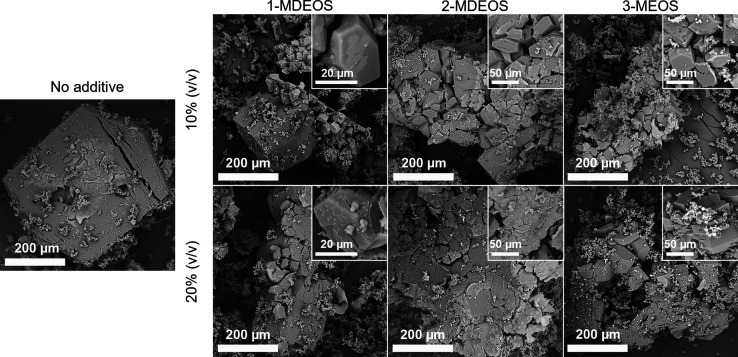
SEM images of HEWL crystals obtained at 70 mg/mL HEWL, 40 mg/mL NaCl, 10% (v/v) TMOS, and different AT ratios.

The effect of 1-MDEOS, 2-MDEOS, and 3-MEOS was further assessed on a broader range of supersaturation. The concentrations of HEWL and NaCl were varied between 50 and 80 and 30–50 mg/mL, respectively, at an AT ratio equal to 20% (v/v). For each combination of protein and precipitant, the promotion of nucleation density appeared evident as the number of methyl groups increased (see Figure S2 of Supporting Information). Moreover, the increase of local supersaturation upon the incorporation of 2-MDEOS appeared to be evident at the lowest protein concentration. Indeed, at 50 mg/mL, elongated (110) HEWL faces were obtained as a consequence of a crystallization environment characterized by lower supersaturation,^30^ but this effect was less pronounced in the presence of 2-MDEOS at an AT ratio of 20% (v/v), indicating a higher supersaturation (see Figure S3 of Supporting Information). Therefore, the control of HEWL nucleation was achieved for a protein concentration between 50 and 80 mg/mL and a NaCl concentration between 30 and 50 mg/mL. In this range, it was possible to tune the number and the size of HEWL crystals by controlling the supersaturation inside the gel without inducing precipitation.

### 3.3 Understanding the Nucleation Ability of Modified Gels

#### 3.3.1 Interaction between HEWL and Additives Free in Solution

After the tuning ability of modified silica gels was proven, experiments in MB were conducted to investigate whether the effect of the additives was completely ascribable to the substitution of silanol groups in the silica network or whether a specific interaction between the additives and the protein occurred. The concentration of additive was selected to preserve the protein-to-additive mass ratio consistent with the CDC experiments, i.e., 12, 11.6, and 13 for 1-MDEOS, 2-MDEOS, and 3-MEOS, respectively. These ratios correspond to the worst-case scenario where the maximum concentrations of protein and additives were involved. Figure 7 displays HEWL crystals produced in the presence of 1-MDEOS. At a ratio of about 12 (Figure 7a), HEWL crystals exhibited the common tetragonal shape, composed of 4 (110) and 8 (101) faces, meaning that the crystallization was not altered by adding 1-MDEOS. Moreover, the nucleation density increased for higher protein and precipitant concentrations coherently with the classical nucleation theory. This outcome was confirmed in the presence of 2-MDEOS and 3-MEOS at the protein-to-additive ratio equal to 11.6 and 13, respectively (data not shown). By contrast, the addition of a superior amount of 1-MDEOS to the crystallization environment (Figure 7b) led to a significant modification of the crystals. For each condition, some protein precipitate was detected, and this could be ascribable to the unfavorable crystallization conditions induced by the high content of 1-MDEOS. Furthermore, HEWL crystals displayed elongated (110) faces, which are typical of low-supersaturated solutions.^30^ Indeed, the precipitation of HEWL could have decreased the concentration of dissolved protein and, hence, the supersaturation of the crystallization environment.

**Figure 7:**
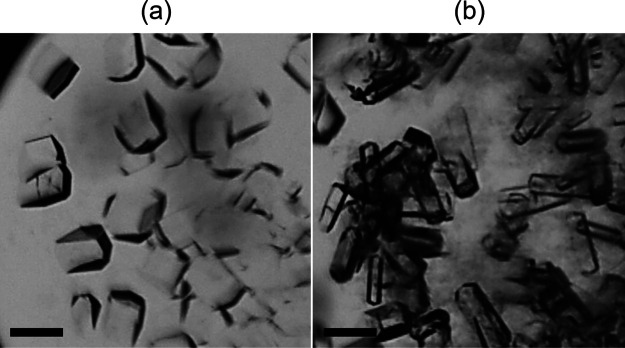
HEWL crystals produced by MB at 37.5 mg/mL protein and 30 mg/mL NaCl+ 1-MDEOS. The mass ratio protein-to-additive was (a) 12 and (b) 0.4. Scale bars refer to 100 μm.

In conclusion, the interactions between the additives and HEWL were negligible for protein-to-additive ratios of about 12 ± 1. However, a marked increase in the additive concentration at a constant protein content completely modified the crystallization environment.

#### 3.3.2 Interaction between HEWL and Additives Immobilized within the Gel Network

To assess the interaction between HEWL and the additives immobilized within the gel network, the release of the protein from HEWL-loaded gels was evaluated with and without the additives. Figure 8a shows the concentrations of HEWL released in the buffer over the gel at different sampling times. Thanks to the establishment of the driving force, the diffusion of HEWL from the gel to the buffer compartment occurs. Compared to the spare TMOS gels, the presence of additives pushed the protein release, and the highest release was achieved in the presence of 2-MDEOS. The total mass of released HEWL at 139 h is reported in Figure 8b. Again, 2-MDEOS allowed for the highest mass of HEWL to be recovered in the buffer, while the poorest (slowest) release was found with the spare gel. Student’s *t* test evidenced a statistically significant difference between the release from the nonmodified gel and the gel added with 20% (v/v) 1-MDEOS (*p* < 0.05), 10% (v/v) 2-MDEOS (*p* < 0.05), 20% (v/v) 2-MDEOS (*p* < 0.001), 10% (v/v) 3-MEOS (*p* < 0.05), and 20% (v/v) 3-MEOS (*p* < 0.001). No statistically significant difference was found between the release measured from 1-MDEOS and 2-MDEOS considering different AT ratios. Instead, the increase in the release from 10% (v/v) to 20% (v/v) 3-MEOS was statistically significant (*p* < 0.05). The comparison for the additive equal to 10% (v/v) AT ratio disclosed a statistically significant difference between 2-MDEOS and 3-MEOS (*p* < 0.05). For an AT ratio equal to 20% (v/v), 2-MDEOS induced a statistically significant different release compared to 1-MDEOS (*p* < 0.001) and 3-MEOS (*p* < 0.05), and a statistically significant difference was detected between 1-MDEOS and 3-MEOS (*p* < 0.05). For further details, the reader is encouraged to refer to Table S1 of Supporting Information. The release trend matched the nucleation trend in CDC and the batch gel. Indeed, the higher protein recovery observed with the modified gels was ascribable to the higher availability of free HEWL macromolecules, i.e., not adsorbed on the gel. However, the releases with 1-MDEOS and 3-MEOS were similar despite the different nucleation densities. This observation may be explained considering the gel structure and chemistry.

**Figure 8:**
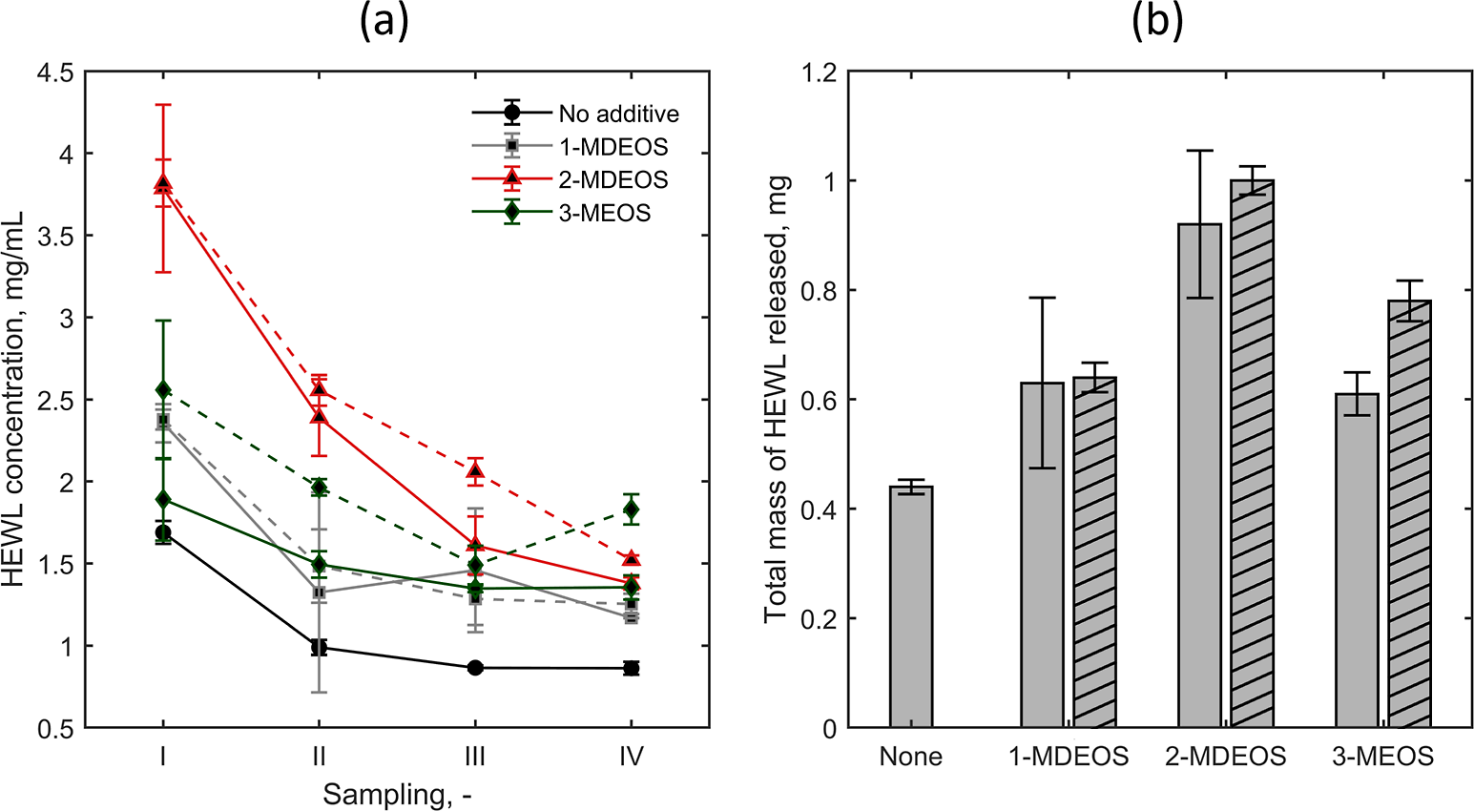
(a) Release profile of HEWL from the gel. 1-MDEOS, 2-MDEOS, and 3-MEOS solid lines refer to 10% (v/v) AT ratio while dashed lines to 20% (v/v). Samplings I, II, III, and IV refer to 24, 43, 67, and 139 h, respectively, after the beginning of diffusion. (b) Total mass of HEWL released from the gel. The filling pattern of 1-MDEOS, 2-MDEOS, and 3-MEOS bars refers to (none) 10% (v/v) AT ratio and (single diagonal) 20% (v/v) AT ratio.

#### 3.3.3 Correlation between Protein Crystallization and the Gel Physicochemical Properties

The interaction between the modified gels and protein macromolecules has been proven to impact crystallization processes, as well as the release of the protein from the gel. We further investigated the underlying mechanism by characterizing some of the gel properties.

The incorporation of methyl groups into the gel network is expected to impact the hydrophobic characteristics of the material. As reported in Table 2, the water CA was the lowest for nonmodified dried gels, indicating a strong hydrophilicity attributable to the presence of Si–OH and Si–O–Si bonds. The hydrophobicity of the gel increased when 1-MDEOS and 2-MDEOS were added to the network. However, when 3-MEOS was used, the hydrophobicity of the silica network was comparable to 2-MDEOS and even lower when the AT ratio was equal to 20. This observation highlights that the hydrophobic character of the gel does not scale linearly with the number of methyl groups in the additive structure.

**Table 2:** Water CAs of Dried Silica Gels

Gel	Additive	AT, -	CA (±SD), °
TMOS 10% (v/v)			24.1 (±3.8)
TMOS 10% (v/v)	1-MDEOS	10	30.6 (±4.1)
TMOS 10% (v/v)	1-MDEOS	20	33.3 (±4.4)
TMOS 10% (v/v)	2-MDEOS	10	74.2 (±3.9)
TMOS 10% (v/v)	2-MDEOS	20	57.9 (±7.1)
TMOS 10% (v/v)	3-MEOS	10	60.4 (±3.9)
TMOS 10% (v/v)	3-MEOS	20	49.1 (±6.4)

Alongside CA, the mechanical properties of the gel were also measured via rheometry. Figure 9 shows the trend of the storage modulus, *G*′, as a function of strain frequency, for nonmodified and modified silica gels. *G*′ was independent of frequency, suggesting a gel-like behavior of the material, as expected. Interestingly, the lowest *G*′ was observed for the nonmodified gel. The incorporation of additives improved the elasticity of the gel and increased the extent of cross-linking. This result was attributed to the modified structure of the gel obtained upon incorporation of the additives. We hypothesize that, in the presence of TMOS alone, the resulting gel is less interconnected due to the scarce accessibility of residual silanol groups participating in condensation reactions after the formation of the primary silica particles. When methylated additives are added to the system, they are readily incorporated into the silica clusters and, due to the increased number of terminal groups not participating in condensation reactions, the resulting structure is more open and the accessibility of silanol groups is improved, resulting in a higher cross-linking degree. This trend holds true for the 1-MDEOS and 2-MDEOS additives. Similar to what had been observed with CA, the addition of 3-MEOS results in a different structure, i.e., *G*′ decreases. This behavior was attributed to the excessive amount of methyl groups, which may have triggered the formation of local hydrophobic clusters and reduced the elasticity of the gel.

**Figure 9:**
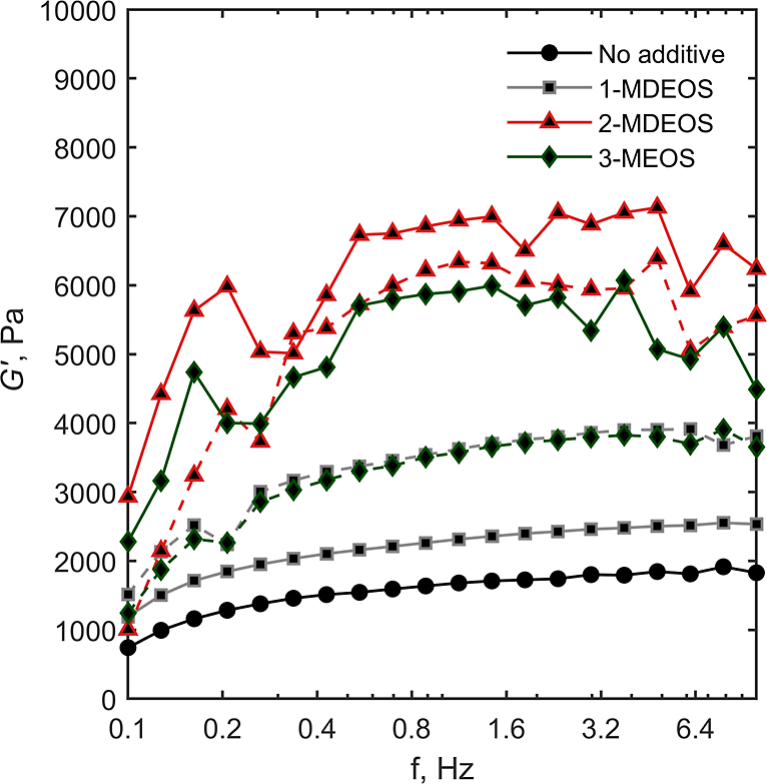
Storage modulus (*G*′) of 5% (v/v) silica gels as a function of strain frequency. 1-MDEOS, 2-MDEOS, and 3-MEOS solid lines refer to 10% (v/v) AT ratio while dashed lines to 20% (v/v).

Hence, we hypothesized that the addition of 3-MEOS dramatically affects the gel network, exposing more hydrophilic groups than expected, thus being more accessible to the protein and increasing the adsorption. Such a finding explains the reduced release of the protein from the corresponding modified gel, as shown in Figure 8a, as well as the saturated nucleation induction effect of gels modified with additives observed both in CDC and batch crystallization.

## 4 Conclusions

In this work, we have reported the use of chemically modified silica gels exposing hydrophobic moieties for the control of crystallization of a model protein, i.e., HEWL. This strategy proved to be successful in overcoming the inhibitory effects of silica gels on nucleation through the simple substitution of silanol groups with methyl groups. Moreover, we have proven that the nucleation-inducing action depended on the number of methyl groups carried by the additives incorporated into the gel network. These results point out that the adsorption of protein macromolecules on the network can be modulated by tuning the gel hydrophobicity and physical properties. In addition, we demonstrate for the first time the possibility of using the batch method to control the crystallization of HEWL in silica gels. This setup leads to uniform and tunable crystal sizes thanks to the establishment of precise supersaturation under a diffusion-controlled mass transport regime.

Growing protein crystals in such gels paves the way for a variety of potential applications. We believe that batch crystallization in modified silica gels represents a versatile platform to obtain high-quality crystals for different applications, ranging from microcrystal slurries for structural determination via XFEL to ultrastable silica–protein composite crystals. In addition, the modulation of the interaction between the silica fibers and the protein macromolecules may be exploited to tune the release kinetics of biotherapeutics supplied as crystalline solid dosage forms.

## Supplementary Material



## Notes

Additional information on the methods, batch crystallization of HEWL in silica gels, and statistical tests on the results of protein release experiments (PDF)

## List of Abbreviations

1-MDEOS: methyldiethoxysilane
2-MDEOS: dimethyldiethoxysilane
3-MEOS: trimethylethoxysilane
AT: additive-to-TMOS ratio
CA: contact angle
CDC: counter-diffusion crystallization
HEWL: hen egg white lysozyme
MB: microbatch
PEG: poly­(ethylene glycol)
SANS: small-angle neutron scattering
SD: standard deviation
TMOS: tetramethoxysilane
XFEL: X-ray free electron laser

## List of symbols

*G*′: storage modulus
