# Supplementary material for: Turning Down the Inhibition Effect of Silica Gels in Protein Crystallization
Source: ACS Appl Mater Interfaces. 2025 Jun 20;17(26):37698–706. doi: 10.1021/acsami.5c07593 (PMC12232274; doi:10.1021/acsami.5c07593)
Supplement: Supplementary file 1 [file am5c07593_si_001.pdf]

Supporting information for

"Turning down the inhibition effect of silica gels in protein crystallization"

Lorena Pasero[1], *Roberto Pisano*[1], *José A. Gavira*[2*], *Fiora Artusio*[1*]

[1] Department of Applied Science and Technology, Politecnico di Torino, 24 corso Duca degli Abruzzi, 10129 Torino, Italy

[2] Laboratorio de Estudios Cristalográficos, Instituto Andaluz de Ciencias de la Tierra (Consejo Superior de Investigaciones Científicas), Avenida de las Palmeras 4, 18100 Armilla, Granada, Spain

*Corresponding authors: fiora.artusio@polito.it, j.gavira@csic.es

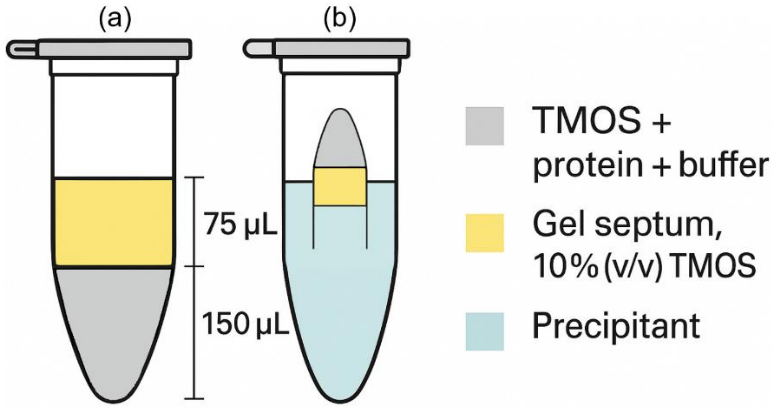

Figure S1. Visual representation of the CDC set-up. (a) A PCR tube was filled with 150 µL of a solution (TMOS, protein, and buffer), and 75 µL of a 10% (v/v) TMOS solution. (b) The open PCR tube was inserted into a 1.5 mL Eppendorf tube® filled with precipitant solution.

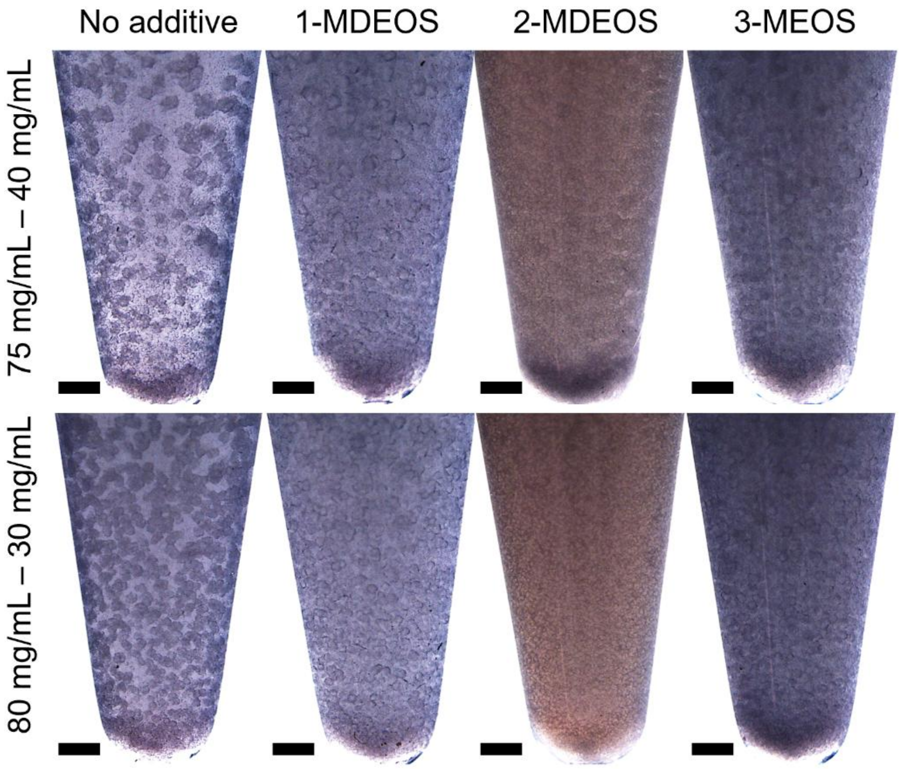

Figure S2. HEWL crystals produced by batch crystallization in silica gel at 10% (v/v) TMOS and an additive-to-TMOS ratio equal to 20% (v/v). Crystals were produced at 75 mg/mL HEWL – 40 mg/mL NaCl and 80 mg/mL HEWL – 30 mg/mL NaCl. Scale bars refer to 1 mm.

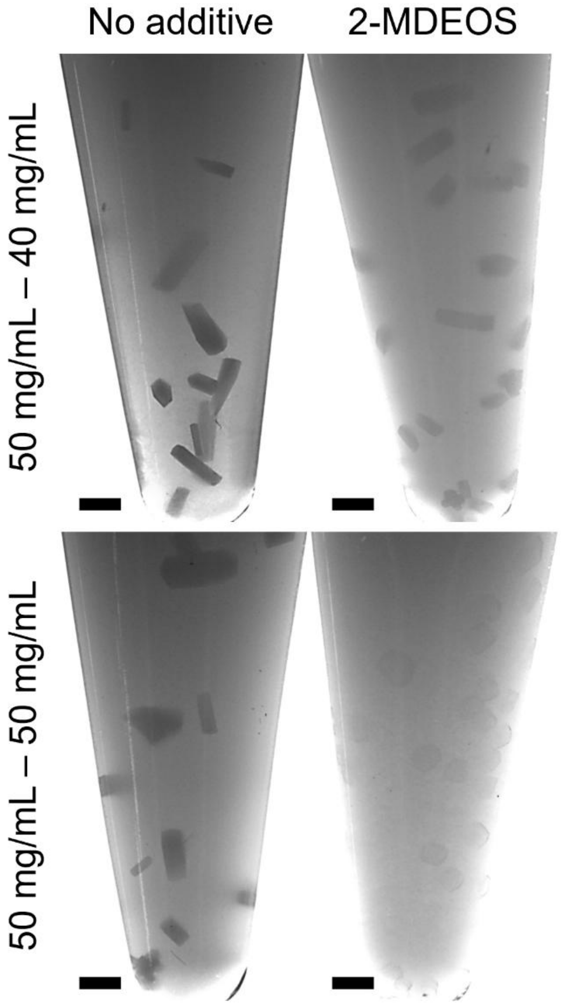

Figure S3. HEWL crystals produced by batch crystallization in silica gel at 10% (v/v) TMOS without additive and at 20% (v/v). Crystals were produced at 50 mg/mL HEWL – 40 mg/mL NaCl and 50 mg/mL NaCl. Scale bars refer to 1 mm.

Table S1. Student's t-test of the total mass of HEWL released at 139 h.

Comparison	Total mass of HEWL released	
	t	p
Comparison for factor: AT ratio within 1-MDEOS		
20 vs 0	-9.574	0.011
10 vs 0	-1.715	0.228
20 vs 10	-0.084	0.941
Comparison for factor: AT ratio within 2-MDEOS		
20 vs 0	-27.417	< 0.001
10 vs 0	-4.956	0.038
20 vs 10	-0.811	0.502
Comparison for factor: AT ratio within 3-MEOS		
20 vs 0	-12.337	< 0.001
10 vs 0	-5.758	0.029
20 vs 10	-4.593	0.044
Comparison for factor: additive within 10% (v/v)		
2-MDEOS vs 1-MDEOS	-1.957	0.189
2-MDEOS vs 3-MEOS	3.094	0.091
3-MEOS vs 1-MDEOS	0.194	0.864
Comparison for factor: additive within 20% (v/v)		
2-MDEOS vs 1-MDEOS	-13.595	< 0.001
2-MDEOS vs 3-MEOS	6.587	0.022
3-MEOS vs 1-MDEOS	-4.456	0.047
